# First study on phenotypic and morphological characteristics of Malaysian Kedah-Kelantan cattle (*Bos indicus*) and method of estimating their body weight

**DOI:** 10.14202/vetworld.2022.728-736

**Published:** 2022-03-26

**Authors:** Mohammed Sirajul Islam, Nurhusien Yimer, Abd Wahid Haron, Faez Firdaus Jesse Abdullah, Mark Hiew Wen Han, Kamalludin Mamat-Hamidi, Hafizah Binti Mohamad Zawawi

**Affiliations:** 1 Department of Veterinary Clinical Studies, Faculty of Veterinary Medicine, Universiti Putra Malaysia, 43400 Serdang, Selangor, Malaysia; 2 Animal Production Research Division , Bangladesh Livestock Research Institute, Savar, Dhaka-1341, Bangladesh; 3 Department of Animal Science, Faculty of Agriculture, Universiti Putra Malaysia, 43400 Serdang, Selangor, Malaysia; 4 Department of Veterinary Services, Pusat Ternakan Haiwan Tersat, 21700 Kuala Berang, Terengganu, Malaysia

**Keywords:** characteristics, Kedah-Kelantan cattle, morphometric, phenotypic

## Abstract

**Background and Aim:**

Indigenous Kedah-Kelantan (KK) cattle are well adapted with distinguished reproductive capabilities; they account for more than 70% of the domestic beef production in Malaysia. The published literature on the phenotypic and morphometric characteristics of KK cattle are sparse and require further improvement. Therefore, this study was aimed to determine the phenotypic and morphometric characteristics of Malaysian KK cattle and method of estimating live body weight (BW).

**Materials and Methods:**

Morphometric and phenotypic measurements were taken from 184 KK cattle (102 males and 82 females) sourced from three regions. Each animal’s color pattern was recorded for their coat, muzzle, face, eyelashes, horns, tail switch, hoof, and legs through visual observation. Length measurements were taken of the body, face, ear, horn, tail, and rump. Several morphological features such as length, width, and girth were measured using a measuring tape, while wither height and hip height were assessed with a measuring scale.

**Results:**

Brown is the predominant coat color in KK cattle (>82%). The overall means of head length, face width (FW), ear length, horn length, wither height, heart girth (HG), body length (BL), and rump length were 42.5±4.5, 17.3±2.9, 19.8±3.1, 9.9±4.4, 104.3±7.1, 127.4±13.2, 98.3±12.3, and 32.4±4.1 cm, respectively. Different morphometric parameters of length, width, and circumference were significantly ( p<0.01) larger in males than females, except for tail length and TG. Correlation coefficient and multiple regression analysis clearly revealed that BL is the best parameter for estimating live BW in KK cattle.

**Conclusion:**

Phenotypic and morphometric measurements in this study showed that Malaysian KK cattle generally possess a brown coat pattern with smaller body size, while BL revealed to be the best parameter to predict BW. The data generated from this study would be useful as baseline data for the identification and selection of KK cattle based on their phenotypical- and morphological-features for further improvement of this breed.

## Introduction

The Malaysian Kedah-Kelantan (KK) cattle are a hardy and well-adapted, tropical, native cattle breed kept for meat production. They are highly fertile with a strong mothering ability [1] and are uniquely, genetically adapted to high temperatures, humidity, and rainfall. In addition, they have high survivability, are resistant to common diseases, and are able to utilize low-quality indigenous forage with minimum housing facility while producing one calf/year [2]. About 90% of these cattle are reared under a traditional farming system with very few amounts of feed supplementation [3,4]. KK cattle are better for lean meat production in comparison to their crossbreeds, as they have a better feed conversion [4]. KK cattle make up most of the domestic beef production in Malaysia, since exotic, imported exotic breeds, crossbreeds, and synthetic breeds are unable to perform to their full potential in Malaysia [5].

The importance of KK cattle in the domestic beef industry in Malaysia has been increasing gradually in recent years to meet the growing demand for beef in Malaysia. KK and their crossbreed cattle are the only pillars available to promote and expand domestic beef production in this country. The potential productive and reproductive characteristics of KK cattle can be used to produce genetically superior animals for commercial purposes through long-term breeding; however, sufficient phenotypic and morphometric information is essential to select superior animals for conservation and breeding to produce a more productive herd [6]. Phenotypic measurements, such as coat color, are important selection criteria for breeding animals because phenotypic measures have a strong relationship with the animal’s ability to cope with environmental stressors [7]. For example, various tick and tick-borne diseases affect more than 80% of cattle and are responsible for great economic losses, but phenotypic traits of the cattle, such as coat color, can improve tick resistance and well control tick-borne diseases [8]. In addition, rural smallholder cattle farmers frequently utilize coat colors to identify individual animals in a herd.

Biometric measurements of body conformation are important part of an animal’s phenotypic characterization. They determine morphometric characteristics and individual animal conformation. A combination of multiple morphometric measurements can provide a superior guide to describe the type and function of domestic animals [8]. Morphometric measurements significantly vary with differences in age and sex, as observed by various researchers in cattle [9,10], horses [11,12], goats [13], and sheep [14]. Breed type and function are considered better indicators of the usefulness of an animal than weight because BW itself is limited without the animal’s associated phenotype and conformation [15]. The prediction of BW based on morphological measurements is an inexpensive method in rural areas that is more practical, quicker, and easier for producers and breeders to implement [16].

In spite of serving as the main contributor to domestic beef production, KK cattle are not yet documented by the Genetic Bureau or by the Food and Agricultural Organization of the United Nations. In addition, knowledge on phenotypic and morphometric characterization of KK cattle through proper investigation has not yet been accomplished, warranting the need to conduct the present study.

The study aimed to determine the phenotypic and morphometric characteristics of Malaysian KK cattle, as well as the morphometric parameters that might be used to estimate their BW.

## Materials and Methods

### Ethical approval

Ethical approval to conduct this study was obtained from the Institutional Animal Care and Use Committee (IACUC), Universiti Putra Malaysia (UPM), Selangor, Malaysia (Ref. No. UPM/IACUC/AUP/RO-96/2018).

### Study period and areas

**Figure-1:**
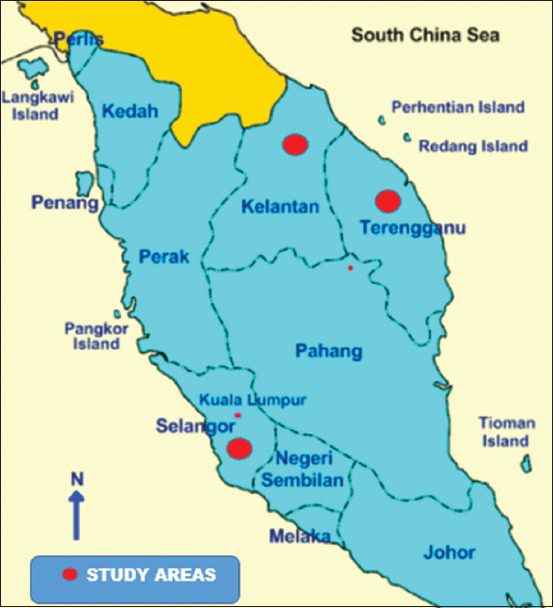
Map of the study areas (www.mapsofworld.com)

This study was conducted from September 2020 to April 2021. This study was conducted at three government cattle breeding farms in different agro-ecological locations in Malaysia as shown in Figure-1. The Pusat Ternakan Haiwan Tersat, 21700 Kuala Berang, Terengganu, Darul Iman, Malaysia (5.0738°N; 103.0127°E), under the department of veterinary services (DVS) maintained a KK purebred cattle breeding herds consisting 978 heads. The weather of this area is influenced by a tropical wet climate without any dry season. The climate is classified as tropical having a significant amount of rainfall throughout the year located 9 m above the sea level. The average annual rainfall, temperature, and humidity are 2498 mm, 26.1°C, and 84.0%, respectively. The Pusat Ternakan Haiwan Pantai Timur of DVS, Tanah Merah, Kelantan, Malaysia (5.8012°N, 102.4766°E), where a breeding herd of purebred KK having a population of more than 500 heads, was maintained. In addition, purebred KK bulls were used from the Taman Pertanian Universiti (TPU), UPM, Serdang, Selangor, Malaysia (2°59′09″ N; 101° 43′51″ E).

### Experimental design

**Figure-2:**
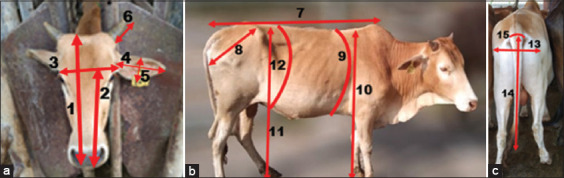
(a-c). Diagram for the measurement of head length (1), face length (2), face width (3), ear length (4), ear width (5), horn length (6), body length (7), rump length (8), heart girth (9), whither height (10), hip height (10), flank girth (12), rump width (13), tail length (14), and tail base (15).

One hundred and eighty-four purebred Malaysian KK cattle (102 males and 82 females) were used for the phenotypic observation and morphometric measurements. The phenotypic characteristics were recorded for all 184 KK cattle. Specifically, we recorded the color of each animal’s coat, face, muzzle, eyelashes, horns, legs, abdomen, and switch tail, as well as the type of hump and dewlap, according to Barth [17]. Ages of the studied animals were calculated from birth records kept at each breeding farm. BW was measured using an animal weighing balance. Fifty-one purebred KK cattle of both sexes (37 males and 14 females) within an age range of 24-36 months were used to measure the morphometric traits by restraining each animal in a service chute in accordance with Food and Agriculture Organization guidelines [18]. The first author performed majority of the measurements from the left side of each animal to avoid bias. All morphometric traits were measured by a measuring tape and measuring scale according to Chandran *et al*. [19]. Table-1 and Figure-2 illustrate the details of the morphological parameters measured and how the measurements were taken.

**Table-1:** Description of the morphological measurements of the Kedah-Kelantan cattle.

Body measurement (cm)	Measurement
1. Head length (HL)	Distance from the nape to the rostral end of the muzzle
2. Face length (FL)	Distance from the widest part of head to rostral end of muzzle
3. Face width (FW)	Distance of the widest points of the head
4. Ear length (EL)	Distance from the root to the end point of ear
5. Ear width (cm)	Distance of the largest points of the ear
6. Horn length (LH)	Distance from the base of horn to the tip.
7. Body length (BL)	The horizontal distance from the point of shoulder to pin bone
8. Rump length (RL)	The horizontal distance from the point of hip to the pin bone
9. Heart girth (HG)	By placing the measuring tape around the animal at the point of smallest circumference just behind the forelegs
10. Wither height (WH)	Distance (vertical) from the bottom of the front foot to the highest point over wither.
11. Hip height (HH)	Distance from the bottom of the hind foot to the highest point between hooks (tuber coxae).
12. Flank girth (FG)	By placing the measuring tape around the animal immediately in front of the hind legs
13. Rump width (RW)	The distance between the points on either side of the animal located at one half of the distance measured from ventral point
14. Tail length (TL)	Distance from the base of the tail proximal end of the first coccygeal bone to the distal end of the last coccygeal bone.
15. Tail girth (TG)	The biggest circumference at the base of the tail

### Management of KK cattle

Pond water was the main source of water at all times for the cattle. The animals were grazed on pastureland under an extensive system with little or no feed supplementation. No housing system was provided, except for a simple shed for vaccinations, deworming, and medications. Palm kernel cake and soya hull were provided to the animals at times when there was a shortage of pasture access due to heavy rainfall or pastureland preparation. All animals were vaccinated against foot and mouth disease in 6-month intervals and against hemorrhagic septicemia in 1 year interval. Deworming was done to all animals every 6 months. The purebred KK bulls at TPU and UPM were provided Guinea grass (*Panicum maximus*) and concentrate feeds under an intensive management system. A natural breeding system was used with different sires having free range in the extensive husbandry system.

### Statistical analysis

The morphological data were statistically analyzed and expressed as percentages, means, standard deviations (SD), and coefficient of variations (CV) using Microsoft Excel 2016, version 16.0.4324.1002 (Microsoft Corporation, USA) The formula of CV= (SD/Mean (m)×100 was used and expressed as a percentage. The Pearson correlation coefficient (r) was used to determine the relationship between the morphological characteristics, age, and animal weight through bivariate correlations using the Statistical Package for the Social Sciences computer software program, version 22, (IBM Corp. NY, USA). The strength of each relationship was considered negligible (r<0.2), low (r=0.2-0.4), moderate (r=0.4-0.7), high (r=0.7-0.9), and very high (r>0.9).

According to Guildford [20], prediction of BW and its relationship with age, and morphometric parameter of head length (HL), WH, heart girth (HG), and body length (BL) were assessed based on model summary (R and R^2^) of the following regression or prediction equation:

Ŷ=Bo+B_1_X_1_+B_2_X_2_+B_3_X_3_+B_4_X_4_+B_5_X_5_+B_6_X_6_

Where, Y=Dependent variable, Ŷ=Predicted value of BW (Y), Bo=Y-intercept, X_1_=age of animals, X_2_=birth weight, X_3_=HL, X_4_=WH, X_5_=HG X_6_=BL, and B_1-6_=regression coefficients

Hypothesis test of regression model: H_0_=B_0_+ɛⅈ; H_A_=B_0_+B_1_X_1_+B_2_X_2_+B_3_X3+B_4_X_4_+B_5_X_5_+B_6_X_6_+ɛⅈ

Hypothesis test of independent variables:

For age: H_0_=B_1=0;_ H_A_=B_1_≠0, for birth weight: H_0_=B_2_=0; H_A_=B_2_≠0, for HL: H_0_=B_3_=0; H_A_=B3≠0, for WH: H_0_=B4=0; H_A_=B_4_≠0, for HG: H_0_=B_5_=0; H_A_=B_5_≠0, and for BL: H_0_=B_6_=0; H_A_=B_6_≠0.

## Results

### Phenotypic observations

**Figure-3:**
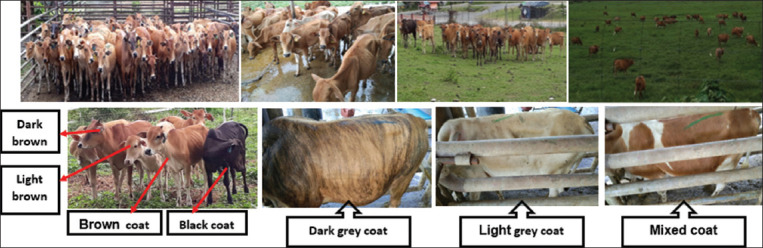
Coat color phenotypes of Malaysian Kedah-Kelantan cattle.

**Figure-4:**
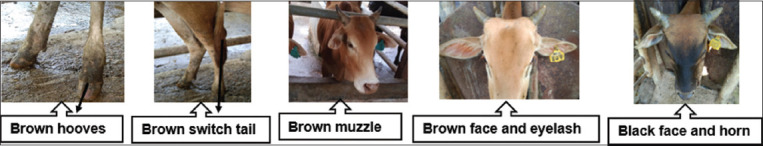
Color phenotypes of hooves, switch tail, muzzle, face, eyelash, and horns in Kedah-Kelantan cattle.

The phenotypic characteristics obtained are presented in Table-2. A wide variation in terms of color pattern was observed: 38.6% light brown; 31.5% brown; 12.5% reddish-brown; 11.4% gray; 2.2% black; and 7% miscellaneous (combination of various colors and mixtures; Figure-2). White spots were seen on parts of the legs and abdomens of a few animals. Skin color was black in about 73.9% of the animals and brown in about 26.1%. Face color was reddish-brown (41.3%), brown (27.7%), light brown (9.8%), black (11.4%), and brown with white spots (9.8%). For muzzle color, 79.4% were black with white circles and 20.7% were light brown. The eyelash color of 69.0% of the animals was black, while 30.9% were brown. Horn’s color was mostly black (88.5%), with 11.5% light brown. Color of the switch tail was mostly black (78.3%), with the remaining 21.7% brown. Hoof colors were similar to switch tail color, with more than 78% black. The leg colors were light brown (77.2%), gray (10.3%), and brown with white pigments (12.5%). Abdominal color was mostly brown (85%). There were 24.5%, 50.5%, and 25.0% of the hornless, short-horned, or medium-horned animals, respectively. The horns of more than 50% of the cattle were short, slightly curled, and pointed in shape. The cattle possessed a small (63.0%) to medium (37.1%) sized hump with an erect and flat orientation. Their dewlap was typically large or medium (70.7%) to small (29.4%) with variations in coat color patterns, as shown in Figure-3. The different colored hooves, switch tail, face, muzzle, horn, and eyelash colors are shown in Figure-4.

**Table-2:** Phenotypic characteristics of Malaysian Kedah-Kelantan cattle (males 102 and females 82).

Variables	Color	n	Percentage
Coat color	Light brown	71	38.6
	Brown	58	31.5
	Dark/reddish-brown	23	12.5
	Gray	21	11.4
	Black	4	2.2
	Non-distinguished	7	3.8
Skin color	Black	136	73.9
	Brown	48	26.1
Face color	Dark brown	76	41.3
	Brown	51	27.7
	Light brown	18	9.8
	Black	21	11.4
	Brown with white spots	18	9.8
Muzzle color	Black	146	79.4
	Light brown	38	20.7
Eyelash color	Black	127	69.0
	Brown	57	31.1
Horn color	Black	123	88.5
	Light brown	16	11.5
Switch tail color	Black	144	78.3
	Brown	40	21.7
Hooves color	Black	144	78.3
	Light brown	40	21.7
Leg color	Light brown	142	77.2
	Gray	19	10.3
	Brown with white	23	12.5
Abdomen color	Brown	157	85.3
	Mixed color	27	14.7
Horn type	Hornless	45	24.5
	Short	93	50.5
	Medium	46	25.0
Hump type	Small size	116	63.0
	Medium size	50	36.9
Dewlap type	Medium size	130	70.7
	Small size	54	29.4

### Morphometric measurements

Table-3 shows the morphometric parameters measured in this study. The CV for the studied parameters varied from 6.85% to 19.3%, except for the horn length . All morphometric values of length, girth, and width were significantly (p<0.01) greater in males than females, except for tail length (TL) and tail base girth (TG). HL in the males and females were 44.1±0.3 and 38.7±1.5 cm, respectively, with a highly significant (p=0.000) difference between the sexes. In addition, face length (FL) was significantly (p=0.000) larger in males than females. Furthermore, face width (FW) was 18.4±0.3 and 14.0±0.7 cm for males and females, with highly significant (p=0.000) differences between them. Ear length also differed significantly (p=0.004) with 20.4±0.6 and 17.9±1.3 cm for males and females, respectively. Similarly, ear width was significantly (p=0.000) larger in males than females. Horn length was almost double in males (11.3±0.5 cm) than females (5.7±1.2 cm) with a highly significant (p=0.000) deviation between them. WH was significantly (p=0.000) greater in males (106.3±0.8 cm) compared to females (98.5±2.5 cm) followed by a similar trend for hip height (HH). HG, flank girth (FG), BL, rump length, and rump width were significantly greater (p<0.01) in males than females with overall means of 127.4, 135.1, 98.3, 32.4, and 21.6 cm, respectively, regardless of sex. On the other hand, no significant difference was found in males and females for TL (p=0.941) and TG (p=0.443).

**Table-3:** Morphometric characteristics of Malaysian Kedah-Kelantan cattle (male 37 and female 14).

Parameters	Sex	Min.	Max.	Range	Mean±SEM	Overall mean	CV%	p-value
Head length (cm)	Male	39	48	09	44.1±0.3	42.5±4.5	10.6	0.000
	Female	24	42	18	38.7±1.5		
Face length (cm)	Male	24	36	12	30.1±0.6	29.5±3.8	13.0	0.003
	Female	22	31	09	27.4±0.7		
Face width (cm)	Male	14	23	09	18.4±0.3	17.3±3.1	17.2	0.000
	Female	10	17	07	14.0±0.7		
Ear length (cm)	Male	10	26	16	20.4±0.6	19.8±3.1	15.4	0.004
	Female	10	24	14	17.9±1.3		
Ear width (cm)	Male	6	13	7	10.8±0.2	10.2±1.9	18.8	0.000
	Female	5	11	6	8.7±3.4		
Horn length (cm)	Male	2	6	14	11.3±0.5	9.9±4.4	44.3	0.000
	Female	0	13	13	5.7±1.3		
Wither height (cm)	Male	96	115	19	106.3±0.8	104.3±7.1	6.9	0.000
	Female	83	106	26	98.5±2.5		
Hip height (cm)	Male	98	116	18	108.1±0.7	106.6±8.3	7.8	0.001
	Female	84	129	45	101.5±3.6		
Heart girth (cm)	Male	117	145	28	132.1±1.2	127.4±13.2	10.3	0.000
	Female	88	129	41	114.1±4.6		
Flank girth (cm)	Male	123	160	37	140.1±1.5	135.1±14.5	10.8	0.000
	Female	96	136	40	119.5±4.3		
Body length (cm)	Male	79	125	46	101.2±1.9	98.3±12.3	12.5	0.001
	Female	79	98	19	88.2±1.8		
Rump length (cm)	Male	29	42	13	33.5±0.5	32.4±4.1	12.6	0.000
	Female	22	36	14	29.1±1.2		
Rump width (cm)	Male	16	31	15	23.7±0.7	21.62±4.4	19.3	0.000
	Female	12	18	06	14.7±0.5		
Tail length (cm)	Male	25	78	53	65.8±1.6	65.25±10.5	16.0	0.941
	Female	61	72	11	65.73±1.1		
Tail base (cm)	Male	14	23	09	18.1±0.4	17.94±2.3	12.9	0.443
	Female	16	20	4	17.5±0.4		

Min.=Minimum, Max.=Maximum, SEM=Standard error of mean, CV=Coefficient of variation

### Relationship between morphometric characteristics to age and BW

Table-4 presents the correlation coefficients (r) of age and BW with the morphometric parameters of the cattle. The age of the cattle was positively and moderately (*r*=.4-0.7) significant (p<0.01) correlations with BW, HL, FW (FL), WH, HH, HG, FG, and BL with moderate (*r*=0.4-0.7) correlations. Age had a positive but a low relationship to FL (*r*=0.2-0.4) BW was had a mostly positive, moderate (*r*=0.4-0.7) and significant (p<0.01) relationship to HL FL, FW, WH, HH, HG, FG, and BL. HL had a positive, moderate (*r*=0.4-0.7) and significant (p<0.01) relationship to FL, FW, WH, HH, HG, FG, and BL. Similarly, FL positively correlated with FW, WH, HH, GH, and FG, but a reverse relationship with BL. Likewise, FW had a positive, moderate, and significant relationship with WH, HH, GH, FG, and BL. In addition, WH had a positive and significantly (p<0.01) higher correlation with HH, GH, FG, and BL. Moreover, there was a positive, moderate, and highly significant relationship with HG, FG, and BL. HG and FG had significant (p<0.01) and moderate correlations with BL in a positive direction.

**Table-4:** Correlation matrix of age and BW with morphometric characteristics of KK cattle.

	BW	HL	FL	FW	WH	HH	HG	FG	BL
Age	0.62**	0.50**	0.21	0.54**	0.66**	0.48**	0.56**	0.54**	0.56**
Body weight	.	0.65**	0.41**	0.68**	0.71**	0.64**	0.66**	0.56**	0.78**
Head length			0.57**	0.61**	0.76**	0.64**	0.76**	0.65**	0.51**
Face length				0.72**	0.34*	0.30*	0.24	0.44**	−0.03
Face width					0.55**	0.51**	0.62**	0.66**	0.42**
Wither height						0.91**	0.81**	0.71**	71**
Hip height							0.75**	0.67**	0.64*
Heart girth								0.85**	0.56**
Flank girth									0.52**

** Significance at the 1% level (p<0.01)

* significance at the 5% level (p<0.05); Btw=Birth weight, BW=Body weight, HL=Head length, FL=Face length, FW=Face width, WH: Wither height, HH=Hip height, HG=Heart girth, FG=Flank girth, BL=Body length, KK=Kedah-Kelantan

### Prediction of BW

There was a high relationship (r=0.89) between BW and age, HL, WH, HG, and BL (Table-5). Combinations of all independent parameters of age, HL, WH, HG, and BL had a significant 80% variance in BW performance (R^2^=0.89). The null hypothesis was rejected (Sig F=0.000) and the regression model fit the data at 0.05 significance. Compared to the other parameters, BL significantly (p=0.000, t=6.32) predicted the BW performance at a 0.05 level of significance followed by age. HL, WH, and HG did not contribute significantly to BW at the same level of significance.

**Table-5:** Multiple regression analysis for variables predicting the body weight of KK cattle.

Model	Unstandardized coefficients		Standard coefficients	T	Significance
	
	B	Std. error	Beta		
Constant	−357.13	58.62	-	−6.09	0.00
Age	3.33	1.23	0.25	2.70	0.01
Birth weight	6.81	1.59	0.38	4.29	0.00
Head length	1.44	1.11	0.16	1.29	0.20
Wither height	0.65	0.79	0.12	0.82	0.42
Heart girth	−0.22	0.37	−0.08	−0.59	0.56
Body length	2.19	0.33	0.72	6.32	0.00

Dependent variable (Y): Body weight; R=0.89; R^2^=0.79; Sig. F=0.000. R=Multiple correlation coefficient (strength of relationship between Y and X’s), R^2^=Coefficient of determination (amount of variance in Y that is explained by X’s), B=Value of regression coefficients of X. T=t-test value and Sig T=Significant value of t-test. KK=Kedah-Kelantan

## Discussion

This study revealed variations in KK cattle for coat color, which indicates that the KK cattle population is heterogeneous. The variability found may be due to preferential selection by each breeding center, since variable coat color is a preferred trait by producers. Coat color also has a direct relationship with the animal’s ability to withstand environmental stressors, such as heat stress, flies, and lice infestation [8]. Brown was the predominant coat color in KK cattle (>82%), followed by a few other color patterns (e.g., gray, black, or a combination of brown with white spots). Coat color variation in animals is greatly influenced by location and climatic conditions [21]. Black (73.9%) and brown (26.1%) skin colors have thus far only been recorded in KK cattle, but no literature are available to compare these findings with the skin color of other cattle. About 80% of KK cattle exhibit a brown face, followed by black (11.4%), and black with brown-white spots (9.9%). Majority (79%) have a black muzzle with some light brown color. Likewise, approximately 70% of the cattle have black eyelashes, with the rest having brown. Majority (88.5%) have a black horn, with only a few with light brown. About 80% of the switch tails were black with some brown. Similar findings were obtained in hoof color. The leg color was mostly (77.2%) brown, followed by gray, or white spots on brown coat. The coat abdomens were mostly (85.3%) brown with other color combinations. More than 75% cattle had small to medium type horns that were pointed. More than 70% of cattle had a hump type that was dominant, followed by small size (63%), while around 70% of the dewlaps were large to medium-sized (Table-2), which clearly indicates the purity of the indigenous KK cattle. There is no published literature on the phenotypic parameters of indigenous KK cattle to compare our findings with, except for coat color phenotype; however, some comparisons may be attempted using indigenous tropical breeds of cattle from other regions of continental Asia. The results of our study were dissimilar to some extent with the findings of Kayastha *et al*. [22], who reported 31.2% brown body coat color in indigenous cattle of Assam, India, followed by white (28.5%), fawn (15.3%), gray (13.5%), black (4.4%), and mixed (7.1%) colors. The prominent color of the tail switch was black (74.5%). Most of the animals had a black muzzle (86.5%), black hooves (84.7%), and black horns (100%). There were >90% solid reddish-brown, >90% black, >80% black, >90% black, >90% black, >80% whitish, and >60% reddish-brown colors in body, hooves, tail switch, eyelid, muzzle, horn, and legs, respectively, in indigenous Pasundan cattle of Indonesia reported by Said *et al*. [23], which is mostly different from our study. These differences might be due to variations in breed, location, and environment. Phenotypic variation in animals is directly related to their respective breed types, geographical location, and climatic conditions [8,24]. The coat, face, muzzle, switch, hooves, muzzle color, and horn types of KK cattle resemble those of Zebu cattle.

The morphometric parameters of length, width, and circumference were significantly (p<0.01) greater in males than females, except for TL and TG. There is no research-based evidence to compare with these results. However, when compared with measurements from other indigenous tropical breeds of the world (Tables-6 and 7) [10,21,22,25-38], other breeds also clearly show sex-based differences. The mean HL of KK cattle was 42.5±4.5 cm irrespective of sex, but male HL was significantly (p=0.000) longer than in females (Table-6). The HLs observed in our study are more or less similar to different indigenous breeds of Asia. The mean BL and HG were 98.3±12.3 and 127.4±3.2 cm in KK cattle with significant variation (p=0.000) between males and females. The BL and HG of various indigenous (*Bos indicus*) cattle from different tropical and subtropical regions of the world are represented in Tables-6 and 7, which show that the measurements obtained here are similar to those of other Asian breeds. The morphometric measurements of cattle from African countries were higher than those of the KK cattle (Tables-6 and 7).

**Table-6:** Differences of morphometric measurements between KK and various indigenous cattle bulls.

Breed	Country	Body length	Heart girth	Wither height	Reference
KK cattle	Malaysia	101.1±1.9	132.1±1.2	106.3±0.8	Our study
Pasundan	Indonesia	122.16±17.5	149.75±14.1	125.75±14.1	[23]
Bali	Indonesia	88.13±13.19	116.54±11.67	94.46±4.30	[25]
Aceh	Indonesia	107.7	138.7	105.6	[26]
Madura	Indonesia	114.5	154.6	116.8	[26]
Pesisir	Indonesia	115.6	131.4	108.4	[26]
Bachaur	India	113.4±0.4	145.7±0.4	115.9±0.4	[20]
Manipuri	India	100.32±0.6	137.69±0.7	106.22±0.5	[27]
Kosali	India	99.89±2.3	137.1±2.8	103.4±2.1	[28]
Ponwar	India	102.5±0.5	158.8±0.9	115.6±0.4	[29]
Assam	India	91.2±0.8	121.69±1.1	98.1±0.8	[22]
Achai	Pakistan	112.12±1.9	134.12±1.95	106.88±1.1	[30]
Siri	Bhutan	108±2.4	170±4.8	138±2.6	[31]
Bulgaria	Ethiopia	119.0±0.2.3	172.9±0.2.8	131.6±0.1.9	[32]
W. Fulani	Africa	152.3±4.6	125.6±4.4	101.1±2.2	[10]
Angoni	Zambia	127.1±4.7	167.3±7.1	135.2±5.3	[33]
Baila	Zambia	132.4±3.8	171.9±5.1	142.2±6.6	[33]
Barotse	Zambia	136.4±6.7	174.8±3.2	135.5±7.3	[33]
Tonga	Zambia	127.0±6.1	171.5±4.6	139.3±5.2	[33]
Mursi	Ethiopia	129.3±1.7	154.6±1.6	121.3±1.9	[32]

KK=Kedah-Kelantan

**Table-7:** Differences of morphometric measurements between KK and various indigenous cows.

Breed	Country	Body length	Heart girth	Wither height	Reference
KK	Malaysia	88.2±1.8	114.1±4.6	98.5±2.5	Our study
Bali	Indonesia	96.501±4.73	122.75±4.57	96.50±6.86	[25]
Bachaur	India	113.4±0.4	145.7±0.4	115.9±0.4	[19]
Manipuri local	India	98.92±1.6	133.8±2.7	104.7±1.7	[21]
Kankrej	India	123.4±0.4	162.6±11.3	124.5±5.6	[35]
Kosali	India	96.56±1.87	120.1±2.1	99.79±2.8	[28]
Ponwar	India	97.1±0.5	140.6±0.5	108.9±0.4	[29]
Assam	India	76.14±0.8	104.61±0.9	85.79±0.7	[22]
RCC	Bangladesh	92.9±0.3	126.3±0.4	94.9±0.3	[36]
Achai	Pakistan	116.4±0.71	138.6±0.64	103.7±0.53	[30]
Siri	Bhutan	98±2	152±1.5	121±2.1	[31]
Bulgaria	Ethiopia	119.0±0.2.3	172.9±0.2.8	131.6±0.1.9	[32]
Kenana	Sudan	133.1±0.2	154.1±0.4	123.6±0.2	[37]
White Fulani	Africa	154.3±7.5	113.7±3.1	100.5±2.7	[10]
Tidili	Morocco	126.1±5.8	144.6±4.1	106.2±3.5	[38]
Oulmes-Zaer	Morocco	138.6±6.2	158.7±5.4	115.0±4.2	[38]
Angoni	Zambia	122.9±3.8	163.4±10.5	131.4±5.0	[33]
Baila	Zambia	126.8±2.3	169.5±9.8	131.5±2.2	[33]
Barotse	Zambia	130.0±3.0	174.5±4.4	134.8±5.0	[33]
Tonga	Zambia	122.8±3.7	168.9±7.6	134.4±5.2	[33]
Mursi	Ethiopia	114.9±0.8	134.3±0.7	104.6±0.9	[34]

KK=Kedah-Kelantan

The significant (p=0.000) difference between males (106±0.8 cm) and females (98.5±2.5 cm) in WH found in our study resembles the variation of bulls and cows of different indigenous breeds (Tables-6 and 7). The results for BL, WH, and HG of various indigenous cattle (*Bos indicus*) in the African regions were greater than the KK bulls and cows. Rump length and width were greater in males than females, indicating that KK bulls would be better suited for beef production than the cows. The results of BL, HG, and WH in this study are comparable to other indigenous cattle, which shows that more attention could be given to improve this breed. Variations in morphometric measurements within and between breeds can influence the adaptability of a breed to a certain production and management system [24,39]. Morphometric measurements in cattle have been shown to differ significantly due to age and sex in various livestock breeds and species [9,10].

BW had a very strong, significant (p<0.01) relationship to HG, HL, and BL. It was also observed that WH had a positive, strong, and significant (p<0.01) relationship to HH, HG, FG, and BL in KK cattle. Due to the very limited available information, our results cannot be compared to those of other studies. The correlation coefficient results showed similar relationships between African goats [40] and indigenous Ankole cattle in Uganda [39]. The correlation coefficient and multiple regression analysis results clearly revealed that BL is the best estimator for measuring live BW in KK cattle. BL is the sole trait that best predicted live weight in KK cattle, although age was also a relatively good proxy trait for estimation. The different morphometric parameters of length, width, and circumference were significantly (p<0.01) greater in males than females, except for TL and TG. It was also observed that WH had a positive, strong, and significant (p<0.01) relationship to HH, HG, FG, and BL in KK cattle.

## Conclusion

It can be concluded that the predominant coat color in KK cattle is mostly brown with a few other color patterns, a large- to medium-sized hump, and a dewlap similar to other Zebu-type cattle. The morphometric parameters of length, width, and circumference were higher in males than females, except for TL and base. This study is the first of its kind conducted to document the phenotypic and morphometric characteristics of indigenous KK cattle based on a properly designed scientific approach. The results of this study could serve as a potential guide to establish the phenotypic and morphometric characteristics of KK cattle for long-term selective breeding. Attempt to cover additional geographical locations was not possible due to the Covid-19 pandemic situation, time, and financial limitation. Hence, further studies should be considered to address this issue.

## Authors’ Contributions

NY, AWH, and MSI: Designed the study. MSI, NY, and AWH: Phenotypic observation and morphometric measurements. MSI: Data analysis. MSI and NY: Drafted and revised the manuscript. FFJA, MHWH, KM and HBMZ: Critically revised the manuscript. All authors have read and approved the final manuscript.
